# Review of the Terminology, Approaches, and Formulations Used in the Guidelines on Quantitative Risk Assessment of Chemical Hazards in Food

**DOI:** 10.3390/foods13050714

**Published:** 2024-02-26

**Authors:** Eva Doménech, Sebastián Martorell

**Affiliations:** 1 Instituto Universitario de Ingeniería de Alimentos Food-UPV, Universitat Politècnica de València, Camino de Vera s/n, 46022 Valencia, Spain; 2 MEDASEGI Research Group, Department of Chemical and Nuclear Engineering, Universitat Politècnica de València, Camino de Vera s/n, 46022 Valencia, Spain; smartore@iqn.upv.es

**Keywords:** health risk, safety margin, cancer risk, margin of exposure, hazard index, hazard quotient

## Abstract

This paper reviews the published terminology, mathematical models, and the possible approaches used to characterise the risk of foodborne chemical hazards, particularly pesticides, metals, mycotoxins, acrylamide, and polycyclic aromatic hydrocarbons (PAHs). The results confirmed the wide variability of the nomenclature used, e.g., 28 different ways of referencing exposure, 13 of cancer risk, or 9 of slope factor. On the other hand, a total of 16 equations were identified to formulate all the risk characterisation parameters of interest. Therefore, the present study proposes a terminology and formulation for some risk characterisation parameters based on the guidelines of international organisations and the literature review. The mathematical model used for non-genotoxic hazards is a ratio in all cases. However, the authors used the probability of cancer or different ratios, such as the margin of exposure (MOE) for genotoxic hazards. For each effect studied per hazard, the non-genotoxic effect was mostly studied in pesticides (79.73%), the genotoxic effect was mostly studied in PAHs (71.15%), and both effects were mainly studied in metals (59.4%). The authors of the works reviewed generally opted for a deterministic approach, although most of those who assessed the risk for mycotoxins or the ratio and risk for acrylamide used the probabilistic approach.

## 1. Introduction

Chemical substances in food play an important role in nutrition and food preservation. However, some of the compounds incorporated or formed along the food chain can endanger the health of consumers [1]. Heavy metals are an example of chemical hazards of environmental origin, which are transferred from soil, water, air, etc., to raw materials [2,3]. Even at low concentrations, these highly toxic substances are non-biodegradable and accumulate in the body’s target organs. Other contaminants are formed in food processing, such as acrylamide, which is produced from the Maillard reaction of asparagine with reducing sugars at high temperatures, or polycyclic aromatic hydrocarbons (PAHs), which form in processing stages, such as drying or smoking and cooking, e.g., grilling, roasting, and frying [4,5,6]. Hazardous compounds can also come from toxins of fungi, plants, and algae [7]. For example, mycotoxins are secondary metabolites of moulds that grow on numerous foodstuffs and can cause serious illnesses such as cancer or liver disease [8]. Chemical hazards can also arise from deliberate use to control crop pests, such as pesticides, or from on-farm veterinary treatments [9], while food contact materials such as formaldehyde, melamine, and phthalates can also be a source of chemicals [10].

Chemicals were the most frequently reported hazards in the Rapid Alert System for Food and Feed in 2021 [11], with pesticides in first place (1231 notifications) at 27% of health-related notifications, and mycotoxins in food in third position (450 notifications). The other most frequently reported chemical hazards were allergens (198 notifications) and food additives and flavourings (176 notifications), mainly due to unauthorised or additive content levels that were too high.

In 1991, the FAO/WHO Conference on Food Standards, Chemicals, and the Food Trade recommended that the Codex Alimentarius Commission (CAC) incorporate risk assessment principles into decision-making processes. Since then, risk analysis has been accepted as an essential part of food safety consisting of three basic elements: risk assessment, risk management, and risk communication [12]. These three components represent essential and complementary parts, which must be integrated and fed back to obtain a practical risk analysis. In 2003, the working draft for applying risk analysis within the CAC framework was compiled. In 2007, guidelines were issued for national authorities. The FAO/WHO meeting in 2009 drafted the harmonisation, updating, and consolidation of principles and methods for risk analysis of chemicals in food, and in 2010 a guide for chemical risk assessment was published [13]. 

Risk management is a decision-making process in which political, social, economic, and technical factors are considered to control a hazard. Thus, risk managers must weigh the possible safety measures, choose the most appropriate, implement them, and monitor their effectiveness. For example, regulating an MRL, defining a safety factor, or banning a pesticide are risk management decisions. The risk analysis process usually begins with risk management, which, as a first step, defines the problem, articulates the objectives of the risk analysis and defines the questions to be answered by the risk assessment.

Risk communication is exchanging information about risk, such as risk assessment findings, risk management decisions, opinions, etc., throughout the risk analysis process between risk assessors, risk managers, consumers, industry, the academic community, and other interested parties.

Risk assessment is defined as the process of calculating the risk to a given target organism, system, or sub-population, including the identification of inherent uncertainties following exposure to a particular agent, plus the relationships between exposure and dose–response adverse effects [14]. This process may be carried out using either a deterministic or probabilistic approach, and the former means that each parameter of the risk equation takes a single value, e.g., the mean, the 95th percentile, the “worst-case”, etc., so that the result would be a single value representing the risk for a single virtual consumer. This method tends to overestimate the risk and does not take into account the uncertainty inherent to the variability of the input data, such as the food consumption, chemical concentrations, physical differences between groups of exposed individuals, etc., [15,16,17,18]. To reduce this drawback, various methods were studied to evaluate the uncertainty-related results of deterministic models [19].

On the other hand, the probabilistic approach allows for the classification of problems and outcomes, the consideration and treatment of the variability, and uncertainty of the input parameters of the risk equation, defined using a probability density function; the calculations are performed using stochastic methods, such as Monte Carlo simulations [20] where the result is a risk probability distribution [17] and permits the application of optimization processes. However, it is pointed out that each probabilistic approach to risk analysis involves deterministic arguments, which help to decide how the likelihood of events will be addressed [21].

The bases for risk assessment and implementation are defined by expert advisory bodies, such as the US Environmental Protection Agency (EPA), the Joint Expert Committee on Food Additives (JECFA), the Food and Drug Administration (FDA), the European Food Safety Authority (EFSA), the European and Mediterranean Plant Protection Organisation (EPPO), the Council of Europe, and the European Centre for Ecotoxicology and Toxicology of Chemicals (ECETOC), and research groups have led to the creation of different approaches and nomenclatures, leading to confusion. 

This review aimed to analyse how quantitative risk assessment is carried out for some of the most critical chemical hazards, such as pesticides, metals, mycotoxins, acrylamide, and PAHs. The document is organised as follows: Section 2 describes the materials and methods used, while Section 3 gives the results: to provide a basis for our findings, the fundamentals of assessing the risks of chemical hazards in food are first introduced, followed by the terms and formulations most frequently used in quantitative risk assessment, and finally we propose suggestions for harmonizing terminology and formulations. Section 4 discusses how the risks of pesticides, metals, mycotoxins, acrylamide, and PAHs are calculated in food, while Section 5 contains the concluding remarks.

## 2. Materials and Methods

The review included papers on the quantitative risk assessment (QRA) of heavy metals, pesticides, mycotoxins, acrylamide, and PAHs with deterministic and probabilistic approaches [17]. A systematic review was conducted considering the PRISMA guidelines, including the search strategy, article selection, and evaluation criteria [22]. 

A total of 348 articles were selected, of which 74 dealt with pesticides, 133 with metals, 63 mycotoxins, 27 acrylamide, and 51 with PAHs. The search strategy was conducted according to the Cochrane protocol [23], in which the best keywords and synonyms were found using MeSH terms and checking keywords in relevant articles and review papers. These terms were used to retrieve all the related articles in the Scopus, Google Scholar, PubMed, and ISI Web of Science international databases. The title, abstract, and keywords were used to apply the selection criteria to the articles published between 2015 and 2022.

The exclusion criteria applied were as follows:

- Books, clinical studies, abstracts, presentations, theses, reviews, commentaries, meta-analyses, conference papers, editorials, and letters to the editor.
- Duplicate content, not written in English, or in non-peer-reviewed journals.
- Articles in which risk was not assessed.
- Acute toxicological endpoint.
- Articles related to environmental risk, soil, water, pollution, and dust.
- Herbal medicines and breastmilk.
- Biomonitoring studies.
- Experimental lab studies to check the influence of treatment conditions, etc.
- Studies with less than five analysed samples.
- Non-marketable products.
- The same authors with the same terminology and risk formulation.

## 3. Results

### 3.1. Background of Risk Assessment of Chemical Hazards in Food

**Figure 1:**
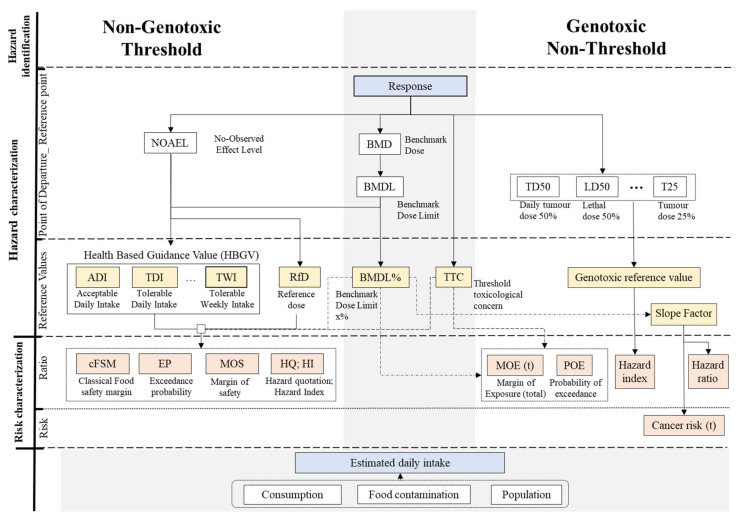
Elements and main parameters of the quantitative risk assessment.

Risk assessment consists of four stages: hazard identification, hazard characterisation, exposure assessment, and risk characterisation. Figure 1 shows an outline of the terms used in the studies reviewed.

Hazard identification decides whether a chemical present in a given food or group of foods has the inherent capacity to cause adverse health effects to consumers and should, therefore, be considered a hazard [24]. All the available data on toxicity and its mode of action (MOA) must be considered to determine the type and nature of an adverse effect. The first key question is to identify whether the compound or its active metabolite reacts covalently with DNA (genotoxic) or whether it has an epigenetic action (non-genotoxic) [25]. In the former case, there is no (threshold) dose that has no potential effect, and DNA damage increases with the dose administered. However, in non-genotoxic cases, it is often assumed that there is an exposure level below which no significant effect will be induced [26]. This difference often determines the choice of the risk assessment methodology. 

To identify chemicals that may have an adverse effect on health, risk assessors can rely on the sources of hazard information published by international organisations. These include the Openfoodtox database, which compiles chemical and toxicological data on chemicals evaluated by EFSA since its inception [27]. CompTox Chemicals Dashboard is the EPA’s computational toxicological research database that provides chemical, toxicological, and exposure information on more than 900,000 chemicals, with more than 300 lists of chemicals, based on their structure or category [28]. Another advisory source for the self-classification of chemicals, which lists more than 54,000 substances, is published by the Danish Environmental Protection Agency [29]. The New Zealand Environmental Protection Agency also publishes detailed information on each chemical’s hazards, classification, studies, and physical properties [30].

Hazard characterisation describes the relationship between the administered dose of a chemical and its MOA or adverse health effect [13]. This relationship is obtained by fitting epidemiological or experimental data obtained from animal or human studies to a dose–response curve [14,31], so that low or no effects to the dose associated with a hazard are identified as the point of departure (POD) or reference point (RP), Figure 1 [32,33]. The no-observable-adverse-effect level (NOAEL) or the lowest-observed-adverse-effect level (LOAEL) are usually taken as the baselines or PODs for non-genotoxic effects. The reference dose (BMD) is another POD derived from the dose–response curve. A predetermined response (BMR) thus identifies a corresponding dose (BMD) or its lower limit (BMDL) defined using the statistical confidence level, typically with 95% confidence, meaning that at a 95% confidence level, the chosen BMR is not exceeded [32,33,34]. 

The BMD or BMDL can be used for non-genotoxic and genotoxic damages. In the first case, it is preferred to the NOAEL or LOAEL for several reasons. Firstly, it is a starting-point estimate based on a NOAEL, which relies solely on identifying a no-effect dose and does not consider the shape of the dose–response curve, and so does not allow the estimation of the probability of response for any dose level. The experimental response observed in the NOAEL may vary between studies, which makes comparison difficult. While NOAEL identification is highly dependent on sample size, a low response rate will have a lower statistical sensitivity to detect small changes, so this type of study tends to generate higher NOAELs. The NOAEL method does not account for variability and uncertainties in the data due to random errors such as animal dosing and response measurements [33]. 

The next step is to specify a reference value (RV), defined as the estimated maximum dose (based on body mass) or concentration of an agent to which an individual can be exposed during a given period without an appreciable risk or a predetermined change in the response rate of an adverse effect. To derive an RV from the NOAEL, this POD must be divided by uncertainty factors to account for interspecies and intraspecies variability, data quality, and other uncertainties arising from the study [35]. Some of the best-known RVs are the health-based guideline values (HBGVs), which include the acceptable daily intake (ADI), developed for food and feed additives, pesticides, and food-contact materials; the tolerable upper intake levels (UL) for vitamins and minerals; the tolerable daily intake (TDI) [35]; the tolerable weekly intake (TWI), in cases where compounds tend to accumulate in the body, such as cadmium, dioxins, or ochratoxin A. The reference dose (RfD) is also an RV. This term is described as an estimate of the daily oral exposure of the human population that is likely to have no appreciable adverse health effects [36]. Another possible RV is the TTC, i.e., threshold of toxicological concern, used for compounds of a known structure, for which exposure is low, but without sufficient experimental data for a fully quantitative risk assessment [37]. Finally, PODs such as the LD 50 (lethal dose to 50% of the population), the T25 (dose that causes a tumour incidence of 25%), and the TD50 (daily dose rate necessary to halve the probability of animals remaining tumour-free at the end of their lives), divided by a safety factor can be used as RVs [38,39]. For example, 50,000 is the uncertainty factor for mycotoxins, equivalent to a risk level of one person in 100,000 inhabitants [40].

To obtain an RV from the BMDL, an increase in response (BMDL%) must be defined, e.g., p = 1%, which means that the incidence (level of response) has increased by 1% relative to the background response. A linear dose–response relationship is assumed at very low doses below the BMDL%, while the increase in the population risk of the effect expressed by the daily dose of a chemical hazard consumed is called the slope factor (SF), Figure 1.

Exposure assessment is the qualitative or quantitative evaluation of the likely intake of a chemical via food [41,42] whose calculation is a function of consumption, concentration of the chemical hazard, and personal weight. As food consumption data are the basis for assessing human exposure, comprehensive information is needed. In this respect, EFSA in the database FoodEx2’s harmonised food consumption data across the European Union (EU). The information is divided by country, food category, age, and sex [43]. The FAO/WHO also provides a chronic individual food consumption database known as CIFOCOss [44]. To estimate dietary exposure to food chemicals, official bodies have developed freely available tools for assessing dietary exposure to food chemicals, such as, DietEx Tool, FAIM for additives, and PRIMO for pesticides, EPA ExpoBox [45,46,47,48]. 

Risk characterisation is the final step in risk assessment and results from the combination of hazard characterisation and exposure assessment. It is defined as the qualitative and/or quantitative estimation of the exposure assessment and the severity of known or potential adverse health effects in a given population [49]. Ratio metrics are applied to assess the risk of non-genotoxic effects, e.g., the hazard quotation, while there are two options for genotoxic effects: to estimate the probability of developing cancer or to assess a ratio, e.g., the margin of exposure (MOE), Figure 1.

### 3.2. Risk Assessment and Hazards

**Figure 2:**
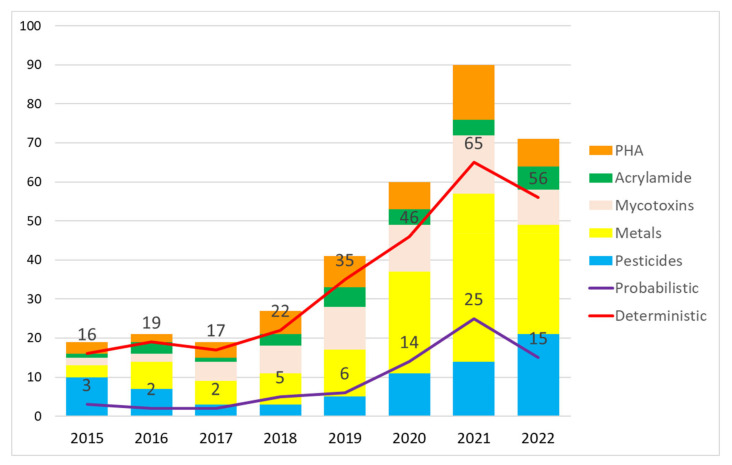
Distribution of the number of papers selected by year of publication and hazard using a deterministic or probabilistic approach to QRA.

Figure 2 shows the number of articles published per year (according to hazards studied) and the trend in using the deterministic or probabilistic approach in risk calculation. The results indicated that the number of articles published increased annually, especially in 2021, possibly due to the COVID-19 pandemic [50,51]. We also found that the deterministic approach was the most frequently used.

Table 1, Table 2, Table 3, Table 4 and Table 5 for pesticides, metals, mycotoxins, acrylamide, and PAHs, respectively, give the information about the utilised risk assessment approach, i.e., deterministic (D) or probabilistic (P); the exposure metric (abbreviations in Table 6 and Table 7), and the equations used by the authors (see Section 3.2.1); the damage, indicating the MOA (G = genotoxic and NG = non-genotoxic) and the RV applied. The last column, on risk characterisation, gives the metric used to calculate the risk (see Table 8, Table 9 and Table 10) and the equation used (see Section 3.2.2).

The articles on pesticides were mainly from China (23.5%), India (10.6%), and Iran (9.4%). The products analysed were mainly on “fruits, vegetables, and legumes”. Almost half the manuscripts studied the risk in adults and children (45.9%), followed by the adult population (38.8%). Exposure was assessed using Equation (1) in 71.8% of cases. The non-genotoxic risk effect was calculated using the ratio with Equations (4) and (5), using the ADI as the reference value at 71.2%, followed by the RfD at 22%. The risk of cancer, Equation (14), was the first option for assessing the genotoxic effect (66.7%). 

**Table 1:** Exposure, damage, and risk characterisation methodology used in pesticide studies.

Reference	D/P	Exposure		Damage (Effect)		Risk Characterisation	
		Metric	Eq.	MOA	RV	Metric	Eq.
Beekeeping products							
[52]	D	ADI	(1)	NG	RfD	HQ, HI	(4), (6)
[53]	D	Exposure	(1)	NG	ADI	HQ, HI	(4), (6)
[54]	D	EDI	(3)	NG	RfD	THQ	(4)
				G	SF	CR	(14)
[55]	D	NEDI	(1)	NG	ADI	%ADI	(6)
[56]	D	EDI	(1)	NG	ADI, RfD	HQ, HI	(4) **, (6)
				G	CPF	CRk, CR	(14) **, (16)
Cereals and bakery products							
[57]	D	EDI	(1)	NG	ADI	HI	(4)
				G	CBC	HR	(12)
[58]	P	CDI	(3)	NG	RfD	HQ, HI	(4), (6)
[59]	D	EDI	(2)	NG	ADI	HQ	(4)
[60]	D	EDI	(1)	G	CBC	HR	(12)
[61]	D	ADD	(3)	NG	RfD	HQ, HI	(4), (6)
				G	CSF	CR	(14)
[62]	D	EDI	(1)	NG	ADI	RQ	(4)
[63]	P	y	(1)	NG	TDI	MOS	(7)
Fats and oils							
[64]	P	CDI	(3)	NG	RfD	THQ, TTHQ	(4), (6)
				G	CSF	CR	(16)
Fish and shellfish							
[65]	D	EDI	(1)	NG	ADI, RfD	HQ	(4)
				G	BMC	HR	(12)
[66]	D	-	-	NG	RfD	THQ	(4) **
[67]	D	EDI	(1)	NG	TDI	HQ	(4)
				G	SF	ILCR	(14) **
[68]	D	EDI	(3)	NG	RfD	THQ	(4)
Fruits, vegs, and legumes							
[69]	D	EADI	(1)	NG	ADI	HRI, CHI	(4), (6)
				G	CBC	HR	(12)
[70]	D	EDI	(1)	NG	ADI	%HQ	(4)
[71]	D	EDD	(1)	NG	ADI	HI	(4)
[72]	D	EDI	(1)	NG	ADI	%cHQ, HI	(4)
[73]	D	EDI	(1)	NG	ADI	HRI, ƩHI	(4), (6)
[74]	D	EDI	(2)	NG	ADI	%RQ	(4)
[75]	D	NEDI	(1) *	NG	ADI	%RQ	(4)
[16]	P	EDI	(1)	NG	ADI	HQ, HI and p-FSM	(4), (6) and (8)
[76]	D	NEDI	(1)	NG	ADI	%ADI	(6)
[77]	D	EDI	(1)	NG	ADI	%HQ	(4)
[78]	D	EDI	(1)	NG	ADI	%HQ, cHI	(4), (6)
[79]	D	Exposure	(1)	NG	ADI	%HQ	(4)
[80]	P	EDI	(3)	NG	RfD	HQ	(4)
[81]	D	-	-	NG	ADI	%ADI	(6)
[82]	D	EDI	(1)	NG	ADI	HHI	(4)
[83]	D	EDI	(1)	NG	ADI	%HQ	(4)
[84]	D	NEDI	(1)	NG	ADI	%ADI, HI	(5), (6)
[1]	D	EDI	(1)	NG	ADI	THQ, HI	(4), (6)
[85]	D	EDI	(2) *	NG	ADI	HQ	(4)
[86]	D	EDI	(1)	NG	ADI	%HQ, cHI	(4), (6)
[87]	D	EDI	(1)	NG	ADI	HRI	(4)
[88]	D	EDI	(1)	NG	ADI	%ADI	(6)
[89]	D	EDI	(1)	NG	ADI	THQ, HI	(4), (6)
[90]	D	EDI	(2) *	NG	ADI	IFS	(4)
[91]	D	EDI	(1)	NG	ADI	%ADI	(6)
[92]	D	EDI	(1)	NG	ADI, RfD	THQ, HI	(4), (6)
[93]	D	EDI	(1)	NG	ADI	HQ, HI	(4), (6)
[94]	D	EDI	(1)	NG	ADI	%HQ, HI	(4), (6)
[95]	D	AFE	(1)	NG	ADI, NOAEL	%ADI and MOE, MOEt	(6) and (7), 10
[96]	P	CDI	(3)	NG	ADI	HQ, HI	(4), (6)
[97]	D	EDI	(1)	NG	RfD	HI	(4) **
				G	SFO	TCR	(14) **
[98]	D	EDI	(1)	NG	ADI	HQ	(4)
[99]	D	EDI	(2) *	NG	ADI	HQ, HI	(4), (6)
[100]	D	EDI	(1)	NG	ADI	%ADI	(6)
[101]	D	EDI	(1)	NG	ADI	THQ, HI	(4), (6)
Milk and dairy products							
[102]	D	EDI	(2)	NG	ADI	THQ, HI	(4), (6)
[103]	D	EDI	(1)	NG	RfD	HR	(4)
				G	BMC	HR	(12)
[104]	D	Exposure	(1)	NG	RfD	HQ, HI	(4), (6)
[105]	D	cEDI	(1)	NG	RfD	HQ, HI	(4), (6)
[106]	P	CDI	(3)	NG	RfD	HQ, HI	(4), (6)
				G	CSF	CR	(14)
[107]	D	EDI	(1)	NG	RfD	HR	(4)
				G	BMC	HR	(12)
[108]	D	EDI	(1)	NG	ADI	-	(4)
				G	CBC	HR	(12)
Miscellaneous							
[109]	D	EDI	(1)	NG	ADI	cHQ, cHI	(4), (6)
[110]	D	EDI	(1)	NG	ADI	HRI	(4)
[111]	D	EDI	(1)	NG	ADI	HQ, HI	(4), (6)
[112]	D	Exposure	(1)	NG	ADI	HQ, HI	(4), (6)
[113]	P	EDI	(1)	NG	RfD	HI	(4)
				G	CSF	LCR	(14)
Nuts, nuts products, and seeds							
[114]	D	EDI	(1)	NG	ADI	%cHQ	(4)
[115]	D	EDI	(1)	NG	ADI	%HQ, cHI	(4), (6)
[116]	P	CDI	(3)	NG	RfD	HQ, THQ	(4), (6)
				G	CSF	CR	(16)
Tea, herbs, and spices							
[117]	D	EDI	(1)	NG	ADI	HQ, HI	(4), (6)
[118]	P	LADD	(1)	NG	ADI	HQ, HI	(4), (6)
[119]	P	CDI	(1)	NG	RfD	%HQ, THQ	(4), (6)
[120]	D	EDI	(1)	NG	ADI	HQ, HI	(4), (6)
Total diet studies							
[121]	P	IEDI	(1)	NG	ADI	HQ, HI	(4), (6)
[122]	D	EDI	(1)	NG	RfD	HQ	(4)
[123]	D	EDI	(1)	NG	ADI	HQ, HI	(4), (6)

* When the exposure units are mg/day per person. ** When the exposure used is Equation (3).

Table 2 shows the results for heavy metals. “Fish and shellfish” were the most studied group of foodstuffs. Most manuscripts came from China (21.4%), Bangladesh (13.5%), and Iran (11.16%) and were mainly focused on the adult population (59.6%). Exposure was assessed using Equations (1) and (3) (approximately 45% for both). Equation (4) was used to characterise the risk in 98.4% of the non-genotoxic studies, and RfD was the most frequently used RV (92%). The cancer risk was assessed using Equation (14) for 95.3% of the cases of studies on the genotoxic effects.

**Table 2:** Exposure, damage, and risk characterisation methodology used in metal studies.

Reference	Elements	D/P	Exposure		Damage (Effect)		Risk Characterisation	
			Metric	Eq.	MOA	RV	Metric	Eq.
Beekeeping products								
[124]	Cd, Cr, Cu, Mn, Pb, Zn	D	EDI	(3)	NG	RfD	THQ, HI	(4), (6)
	Cd, Cr				G	CPS	TCR	(14)
[52]	Mn, Cu, Zn	D	ADI	(1)	NG	RfD	HQ, HI	(4), (6)
[125]	Fe, Ni, Cu, Zn, Pb	D	ADI	(3)	NG	RfD	HQ, HI	(4), (6)
	Cr, Cd, As, Ni				G	SF	CR, CRt	(14), (16)
[126]	Pb	D	DIM	(1)	NG	RfD	THQ, HI	(4) **, (6)
	Pb		CDI	(3)	G	CSF	ILCR	(14)
[54]	Pb, Cd, As, Hg, Cu, Zn, Ni	D	EDI	(3)	NG	RfD	THQ	(4)
	Pb, Cd, As, Ni				G	SF	CR	(14)
[127]	Hg, Cd, V, Cr, Ni, Cu, As, Sb, Pb, Ba, Mn	D	EDI	(3)	NG	RfD	THQ, HI	(4), (6)
	Ni, Cr, Pb, As, Cd				G	CSF	LTCR, LTCRtot	(14), (16)
Beverages								
[128]	Cd, Co, Cr, Cu, Fe, K, Mn, Na, Ni, Pb, Zn	D	EDI	(1)	NG	RfD	THQ, TTHQ	(4) **, (6)
Cereals and bakery products								
[129]	As, Cd, Pb	D	-	-	NG	PTWI, TWI	HQ, HI	(4), (6)
					G	CSF	CR	(14)
[130]	As, Cd, Fe, Ni, Pb	D	DIM	(3)	NG	RfD	HQ, HI	(4), (6)
	As, Cd, Ni, Pb				G	SF	CR, TCR	(14), (16)
[131]	Al, As, Cd, Cu, Mo, Pb	P	EDI	(3)	NG	RfD	HQ, HI	(4), (6)
	As				G	SF	CR	(14)
[132]	Zn, Cu, Cd, Pb, As, Al	D	EDI	(3)	NG	RfD	THQ, TTHQ	(4), (6)
[133]	Cd, Cr, Pb, Zn, Ni, Cu, Hg, As	P	EDI	(3)	NG	RfD	THQ, TTHQ	(4), (6)
	Cd, Cr, Pb, Ni, As				G	CSF	ILCR, TCR	(14), (16)
[134]	Cd, As, Sn, Pb, Hg	D	EDI	(3)	NG	RfD	THQ, HI	(4), (6)
	Pb, As, Cd				G	CSF	TR, TRt	(14), (16)
[135]	As, Zn, Fe, Cu	D	ADD	(1)	NG	RfD	HQ	(4)
	As		EDI	(3)	G	CSF	ILCR	(14)
[136]	Pb, Cd	D	EDI	(1)	NG	RfD	THQ	(4) **
[137]	Cd, Cr, Pb, Cu, Fe, Mn, Zn	D	DED	(1)	NG	RfD and ADI	HQ, HI and R	(4), (6) and (4)
	Pb	D	EDI	(1)	G	CSF	CR	(14)
[63]	As, Cd, Pb	P	y	(1)	NG	PTDI	MOS	(7)
[138]	Ni, Pb, Zn, Cd, Cr, Cu, Mn, As,	D	ADD	(3)	NG	RfD	HQ, HI	(4), (6)
	As, Cr, Ni				G	CSF	ILCR	(14)
[139]	As, Al, B, Ca, Cd, Cr, Cu, Fe, Hg, K, Mg, Mn, Na, Ni, Pb, Se, Zn	D	EDI	(1)	NG	RfD	THQ, HI	(4), (6)
Coffee, cocoa, and preparations								
[140]	Cd, Pb	D	EDI	(1)	NG	TWI, BMDL, RfD	%TWI, %BMDL, THQ, HI	(5), (4), (6)
	Cd, Pb		CDI	(3)	G	CSF	CR	(14)
Fats and oils								
[141]	Pb, Cu, Cd, Cr, As, Zn	D	CDI	(3)	NG	RfD	THQ, HI	(4), (6)
	As, Cd, Cr, Pb				G	CSF	ILCR, ƩILCR	(15), (16)
[142]	Pb, As, Cd, Cr	P	-	-	NG	RfD	THQ, TTHQ	(4) **, (6)
					G	CFS	ILCR	(14) **
Fish and shellfish								
[143]	As, Cd, Hg, Pb	D	EDI	(3)	NG	RfD	THQ, TTHQ	(4), (6)
[144]	Cd, Cr, Cu, Fe, Mn, Pb, Zn	D	EDI	(3)	NG	RfD	THQ, TTHQ	(4), (6)
	Pb				G	CSF	CR	(14) **
[145]	Al, Sn, Zn	D	EDI	(1)	NG	RfD	HRI	(4)
[146]	As, Cr, Cd, Pb	D	EDI	(1)	NG	RfD	THQ, HI	(4) **, (6)
	As, Cr, Cd, Pb				G	CSF	CR	(14) **
[147]	Hg	D	EWI	(1)	NG	PTWI	%PTWI	(5)
[148]	As, Cd, Cr, Cu, Fe, Hg, Mn, Pb, Zn.	D	EDI	(1)	NG	RfD	THQ, HI	(4) **, (6)
	As, Cd, Pb				G	CPS	TR	(14) **
[149]	Cd, Cr, Fe, Ni, Zn, Mn, Pb	D	-	-	NG	RfD	THQ, HI	(4) **, (6)
	Cd, Cr, Ni, Pb				G	CSF	TR	(14) **
[150]	As, Cd, Cr, Cu, Hg, Mn, Ni, Pb, Zn	D	EDI	(1)	NG	RfD	THQ, HI	(4) **, (6)
	Cd, Pb				G	CPSo	TR	(14) **
[151]	As, Cd, Hg, Pb	D	-	-	G	LD50	RI	(11)
[152]	As, Cd, Cu, Cr, Pb, Zn	P	EDI	(1)	NG	RfD	THQ, HI	(4) **, (6)
	As, Cd, Cr, Pb				G	CSF	Risk, Ʃrisk	(14) **, (16)
[153]	Al, As, Cd, Cr, Fe, Cu, Hg, Ni, Pb, Zn	D	EDI	(3)	NG	RfD	THQ, HI	(4), (6)
	As, Cd, Cr, Ni, Pb				G	SF	CR	(14)
[154]	As, Cd, Hg, Pb	D	-	-	NG	RfD	THQ, TTHQ	(4) **, (6)
[155]	As, Cd, Co, Cr, Hg, Mn, Ni, Pb, Zn	D	-	-	NG	RfD	THQ, HI	(4) **, (6)
[156]	Hg, Pb, Cd	D	EDI	(3)	NG	RfD	THQ, TTHQ	(4), (6)
[157]	Hg, Pb, Cd, Ni, Cr, As, Sn	P	EDI	(3)	NG	RfD	THQ, TTHQ	(4), (6)
[158]	Hg	D	EWI	(1)	NG	PTWI,	%Risk	(5)
	As, Pb, Hg, Cd, De, Sn, Zn, Cr, Fe, Co, Ni, Al					RfD	THQ	(4) **
[159]	Co, Cu, Ni, Pb, Zn	P	EDI	(3)	NG	RfD	THQ, TTHQ	(4), (6)
[160]	Cu, Fe, Hg, Zn, Pb	D	I	(3)	NG	RfD	TS	(4)
	Pb				G	SF	CR	(14)
[161]	Ag, As, Be, Cd, Co, Cr, Cu, Fe, Mn, Mo, Ni, Sn, Pb, Zn	D	EDI, EWI	(1)	NG	RfD, PTWI	THQ, TTHQ	(4) **, (6)
	iAs, Cr, Ni, Pb		CDI	(3)	G	CSF	ILCR	(14)
[162]	As, Cd, Co, Cr, Hg, Pb	D	DIM	(1)	NG	RfD	HRI, HI	(4), (6)
[163]	Mn, Fe, Co, Ni, Cu, Zn	D	EDI	(1)	NG	RfD	THQ, HI	(4) **, (6)
	Ni				G	CPS	TR	(14) **
[164]	Cd, Cr, Mn, Ni, Zn	D	EDI	(1)	NG	RfD	THQ, HI	(4), (6)
	Mn, Zn, Ni, Pb, Cr, Cd				G	CSF	CR	(14)
[165]	As, Cd, Cr, Pb	D	EDI	(1) *	NG	RfD	THQ	(4) **
	As, Cd, Cr, Pb				G	CSF	CR	(14) **
[166]	Hg, Cu, As, Cd, Pb	D	EDI	(1)	NG	RfD	THQ, HI	(4) **, (6)
	iAs				G	CPSo	TR	(14) **
[167]	As, Cr, Cu, Fe, Mn, Ni, Sn, Pb, Zn	D	EDI	(1) *	NG	RfD	THQ, TTHQ	(4) **, (6)
	As, Pb				G	CSF	CR	(14) **
[168]	Pb, Cu, Cr, Zn, Cd, As, Hg	P	EDI	(1)	NG	RfD	THQi, THQs	(4) **, (6)
	As, Pb				G	OSF	R	(14) **
[169]	Zn, Mn, Cu, Ni, Cr, Cd, Pb	D	EDI	(1)	NG	RfD	THQ	(4) **
[170]	As, Cd, Pb, Cr, Cu, Ni Zn	D	-	-	NG	RfD	THQ, TTHQ	(4) **, (6)
	As, Cr, Pb				G	CSF	CR	(14) **
[171]	Pb, As, Mn, Fe, Zn, Ni, Cr	D	EDI	(3)	NG	RfD	HQ, HI	(4), (6)
	Ni, As, Pb, Cr, Cd				G	CSF	TCR	(14)
[172]	Cr, Cu, Fe, Pb, Cd	D	EDI	(1) *	NG	RfD	THQ, TTHQ	(4) **, (6)
	Pb				G	CSF	CR	(14)
[173]	Zn, Pb, Cu, Cd, Cr	D	EDI	(1)	NG	RfD	THQ	(4) **
[174]	Cr, Pb, Fe, Zn Ni	D	EDI	(1)	NG	RfD	HQ, HI	(4) **, (6)
	Cr, Ni, Pb				G	CSF	CR	(14) **
[175]	Hg, Cd, Pb	D	EDI	(3)	NG	RfD	THQ, HI	(4)
	Cd				G	CSF	CR	(14)
[176]	Pb, Cd, Hg, As, Al, Fe, Zn, Cu, Ni, Co, Cr	D	EWI	(1)	NG	RfD	THQ, HI	(4) **, (6)
[177]	As, Cd, Co, Cr, Mn, Mo, Ni, Pb, Se	D	EDI	(1)	NG	RfD	THQ, HI	(4) **, (6)
	As, Cr, Ni, Pb				G	CSF	CR	(14) **
[178]	As, Cd, Pb, Hg	D	PTWI	(1)	NG	RfD	HQ	(4) **
	As, Pb				G	CSF	CR	(14) **
[179]	Cr, Mn, Cu, Zn, Pb, Co, Rb, V	D	EDI	(1)	NG	RfD	THQ, HI	(4) **, (6)
	Cr, Pb				G	CSF	CR, TCR	(14) **, (16)
[180]	Cd, Cu, Pb, Zn	D	-	-	NG	RfD	THQ, HI	(4) **, (6)
[181]	Cr, Ni, Cu, Zn, As, Cd, Pb	D	EDI	(1)	NG	RfD	-	(4)
	As, Pb				G	CSF	TR	(14) **
[182]	As, Cd, Co, Cr, Cu, Mn, Ni, Pb, Se, Zn	D	EDI	(3)	NG	RfD	THQ, TTHQ	(4), (6)
	iAs				G	CSF	CR	(14)
[183]	Zn, Cd, Mn, Cu, Cr, Pb, Fe, Co	D	EDI	(1)	NG	RfD	THQ, HI	(4) **, (6)
	Pb, Cr, Cd				G	CSF	CR	(14) **
[184]	Ni, Zn, Cu, Cr, Cd, Pb	D	EDI	(3)	NG	RfD	THQ, HI	(4), (6)
	Ni, Cr, Cd, Pb				G	CPS	TR	(14) **
[185]	Zn, Cu, Cr, Pb, Cd, Hg.	D	EDI	(1)	NG	RfD	THQ	(4) **
	Cd, Cr, Pb				G	CSF	TR	(14)
[186]	As	D	EDI	(1)	NG	RfD	HQ, THQ	(4), (6)
					G	CSF	CR	(14)
[187]	As, Cd, Co, Cr, Cu, Hg, Mn, Ni, Pb, Se, V, Zn	D	EDI, EWI	(1)	NG	RfD	THQ, TTHQ	(4) **, (6)
[188]	Hg, Cd, Pb, V, Ni, Co, Cr, Cu, Zn	D	EDI	(1)	NG	RfD	HQ, HI	(4) **, (6)
	Cd, Pb, Ni, Cr		CDI	(3)	G	CSF	ILCR	(14)
[189]	As, Cr, Cd, Pb	D	EDI	(1)	NG	RfD	THQ	(4) **
	As				G	CSF	CR	(14) **
[190]	As, Cd, Cr, Cu, Li, Hg, Fe, Pb, Zn	D	EWI	(1)	NG	PTWI, RfD	THQ, HI	(4) **, (6)
	As, Cd, Pb				G	CSF	CR	(14) **
[191]	Pb, Cd, Cr, As, Hg	D	EDI	(1) *	NG	RfD	THQ, TTHQ	(4) **, (6)
	Pb, As				G	CSF	CR	(14)
[192]	Cr, Mn, Ni, As, Se, Cd, Hg, Pb	D	EDI	(3)	NG	RfD	THQ, HI	(4), (6)
[193]	Pb, Cd, Hg, As, Cr	P	EDI	(3)	NG	RfD	HQ	(4)
	Pb, Cd, As, Cr				G	SF	ILCR	(14)
[194]	Al, V, Cr, Fe, Co, Zn, Cu, Cd Pb	D	-	-	NG	RfD	THQ, TTHQ	(4) **, (6)
[195]	Cu, Pb, Zn, Fe, Mn, Cd, Cr, Ba, As	D	EDI	(1)	NG	RfD	THQ	(4) **
	As, Pb				G	CSF	CR	(14) **
[196]	Cr, As, Cd, Pb, Zn, Cu, Ni, Hg	D	-	-	NG	RfD	THQ, TTHQ	(4) **, (6)
[197]	As, Cr, Cd, Hg, Cu, Zn, Pb, Fe	D	EDI	(1)	NG	RfD	THQ, TTHQ	(4) **, (6)
[198]	As, Cd, Cr, Cu, Hg, Pb	D	-	-	NG	RfD	THQ, HI	(4) **, (6)
	As, Cd, Pb				G	CSF	TR	(14) **
[199]	Pb, As, Cd, Cr, Zn, Cu, Mn, Ni	D	EDI	(1)	NG	RfD	THQ	(4) **
	iAs				G	CSF	THQcarcin.	(14) **
Fruits, vegs, and legumes								
[200]	Cd, Cr, Co, Cu, Fe, Pb, Zn	D	EDI	(1) *	NG	RfD	HRI and THQ, HI	(4) and (4) **, (6)
	Cd, Cr, Pb				G	CPS	TCR	(14) **
[201]	Al, As, Cd, Pb	D	EDI	(1)	NG	RfD	THQ, HI	(4) **, (6)
	As				G	CPSo	TCR	(14) **
[202]	Cd, Cu, Hg, Pb, Se, Zn	D	DIM	(1)	NG	RfD	HRI	(4)
[203]	Cd, Co, Cr, Cu, Fe, Li, Mn, Pb, Zn	D	-		NG	RfD	HRI and THQ, HI	(4) and (4) **, (6)
	Cd, Cr, Pb				G	CSF	TCR	(14) **
[204]	As, Cd, Cr, Cu, Pb, Zn	D	-	-	NG	RfD	THQ, HI	(4) **, (6)
[205]	Cu, Ni, Zn	D	EDI	(1)	NG	RfD	THQ, TTHQ	(4) **, (6)
[206]	Cd, Cr, Cu, Fe, Ni, Pb, Zn	D	EDI	(1)	NG	RfD	THQ, HI	(4), (6)
[207]	Hg, Pb	D	-	-	NG	RfD	THQ, HI	(4) **, (6)
[18]	As, Cd, Cu, Pb, Zn	P	EDI	(3)	NG	RfD	THQ, HI	(4), (6)
[208]	As, Cd, Pb, Cu Zn	P	EDI	(1)	NG	RfD	THQ, TTHQ	(4) **, (6)
	As, Pb				G	SF	TR	(14) **
[209]	Cd, As, Pb	D	EDI	(3)	NG	RfD	THQ, HI	(4), (6)
	Cd, As, Pb				G	CPS	CR	(14)
[210]	As, Pb, Cd	P	EDI	(1)	NG	PTDI	%HQ	(5)
[211]	Fe, Zn, Mn, Cu, Pb, Cr, As, Co, Ni, Cd, Hg	P	CDI	(3)	NG	RfD	THQ, TTHQ	(4), (6)
	Pb, As, Ni, Co, Cd				G	CSF	CR	(14)
[212]	Cd, Pb, As, Hg, Cr	D	EDI	(1)	NG	RfD	THQ, TTHQ	(4) **, (6)
	Cd, Pb, As				G	CSF	CRi, CR	(14), (16)
[213]	Cd Pb Cr Cu Ni Zn	D	CDI	(3)	NG	RfD	HQ, HI	(4), (6)
	Cd, Cr, Pb				G	SF	CR, TCR	(14), (16)
[214]	As, Al, Cd, Co, Cr, Cu, Fe, Hg, Mn, Mo, Ni, Pb, Zn	D	EWI, EDI	(1)	NG	RfD	THQ, TTHQ	(4) **, (6)
	As, Cd, Cr, Pb				G	CSF	CR, ILCR	(14) **, (16)
[215]	Al, As, Cd, Co, Cu, Fe, Mn, Pb, Cr(VI), Ni, Hg, Zn	D	CDI	(3)	NG	RfD	THQ, HI	(4), (6)
	As, Pb, Cr(VI), Cd, Ni				G	CSF	CR, CCR	(14), (16)
[216]	Mg, Ca, K, P, Na, Cr, Mn, Fe, Ni, Cu, Zn, Mo, As, Cd, Pb	D	EDD	(3)	NG	RfD	HQ, HI	(4), (6)
[217]	Pb, Cd, Cr Ni	D	DIR	(1)	NG	RfD	THQ, TTHQ	(4) **, (6)
[218]	Al, As, Ca, Cd, Cr, Cu, Fe, K, Mg, Mn, Ni, Pb, Zn	D	EDI	(1)	NG	RfD	THQ, HI	(4) **, (6)
[219]	Cd, Cu, Cr, Pb	D	EDI	(2)	NG	RfD	HQ, HI	(4), (6)
	Cd, Cr, Pb				G	CSF	CR, TCR	(14), (16)
[220]	As, Cd, Cr, Hg, Pb	D	-	-	NG	RfD	HQ, HI	(4) **, (6)
	As				G	SF	CR	(14)
[221]	As, Cd, Hg, Pb	P	EDI	(3)	NG	RfD	THQ, TTHQ	(4), (6)
	As				G	CSF	CR	(14)
[222]	As, Cd, Pb, Cr, Mn, Ni, Cu, Zn	D	EDI	(1)	NG	RfD	THQ, HI	(4) **, (6)
	As, Pb				G	Csfo	TR	(14) **
[223]	Cd, Pb	D	EDI	(3)	NG	RfD	HQ, HI	(4), (6)
	Pb				G	SF	ELCR	(14)
[96]	As, Pb, Cd, Cr, Cu, Fe, Hg, Ni, Pb	P	CDI	(3)	NG	ADI	HQ, HI	(4), (6)
	As, Pb				G	CSF	ILCR	(15)
[224]	Cu, Zn, Cr, Ni, Cd, As, Pb Hg	D	EDI	(3)	NG	RfD	THQ, HI	(4), (6)
	Cr, As Pb				G	CSF	CR	(14)
Tea, herbs and spices								
[225]	As, Cd, Pb	D	ADI	(1)	NG	RfD	HQ, HI	(4), (6)
[226]	As, Cd, Hg, Pb	D	EDI	(1)	NG	RfD	THQ, TTHQ	(4), (6)
[227]	Cd, Pb, As, Mn, Ni, Cr	D	EDI	(3)	NG	RfD	THQ, HI	(4), (6)
[228]	As, Cd, Cr, Cu, Hg, Ni, Pb, Zn	D	ADI	(3)	NG	RfD	HQ, THQ	(4), (6)
	As, Cd, Cr, Pb				G	SF	Risk, Riskt	(14), (16)
[229]	Cd, Cr, Cu, Pb, Zn	D	EDI	(1)	NG	RfD	THQ, HI	(4), (6)
[230]	As, Cd, Hg, Pb	D	EWI	(1)	NG	RfD	THQ, TTHQ	(4) **, (6)
	As, Cd, Pb				G	CSF	CR	(14) **
[231]	Cr, Co, Ni, Cu, Zn, Cd, Pb	D	ADI	(3)	NG	RfD	HQ, HI	(4), (6)
	Cr, Cd				G	SF	CR	(14)
Meat and meat products								
[232]	Cd, Cr, Cu, Hg, Pb, Zn	D	EDI	(1)	NG	RfD	HQ, HI	(4) **, (6)
	Cd, Pb	D	EDI and CDI	(1) and (3)	G	BMDL and CSF	MOE and ILCR	(9) and (14)
[233]	As, Fe, Cu, Zn, Mn, Ni, Sr, V, Al, Cr, Cd, Pb	D	EWI	(1)	NG	RfD	THQ, TTHQ	(4) **, (6)
	As, Cd, Cr, Pb				G	CsF	CR	(14) **
Milk and dairy products								
[234]	As, Cd, Cr, Cu, Fe, Hg, Mn, Ni, Pb, Se, Zn	D	EDI	(1)	NG	RfD	THQ, HI	(4), (6)
[105]	Cd, Pb, Cu, Zn	D	cEDI	(1)	NG	RfD	HQ, HI	(4), (6)
Miscellaneous								
[235]	As, Cd, Cu, Pb, Zn	D	D	(3)	NG	RfD	HQ, HI	(4), (6)
	As, Cd, Pb				G	SF	CR	(11)
[236]	As, Cr, Pb, Ni, Cu, Mn	P	EDMI	(1)	NG	RfD	THQ, TTHQ	(4) **, (6)
	As, Pb				G	CSF	TCR	(14) **
[237]	Cr, Ni, Cu, As, Cd Pb	D	EDI	(1)	NG	RfD	THQ	(4) **
	As, Pb				G	CSFo	TR	(14) **
[238]	Pb, As, Cd, Hg	D	EDI	(1)	NG	RfD	THQ, TTHQ	(4) **, (6)
	As				G	SF	CR	(14)
[239]	As, Al, Cd, Cr, Cu, Hg, Mn, Pb, Se, Zn	D	DIM	(1)	NG	RfD	THQ, HI	(4) **, (6)
[240]	As Mn Mo Co Zn Hg Pb Ni Cr Se Cd Al Cu Ag Fe	P	EDI	(3)	NG	RfD	THQ, HI	(4), (6)
[241]	As, Cd, Hg, Tl, Pb, U, Cr, Mn, Fe, Ni, Cu, Zn, Se	D	EDI	(1)	NG	RfD	THQ, HI	(4), (6)
	As				G	CSF	TR	(14)
[242]	Cu, Zn, TAs, iAs, MeHg, Se, Cd, Pb	P	ADD	(3)	NG	RfD	HQ, HI	(4), (6)
	iAs				G	CSF	ILCR	(14)
[243]	Cd, Cr, Pb, Hg, As	P	EDI	(1)	NG	RfD	THQ, TTHQ	(4) **, (6)
Prepared dishes and snacks								
[244]	Cd, Pb, Cr, Mo, Co, Ni, As	P	EDI	(1)	NG	NOAEL	MOE	(7)
Total diet studies								
[245]	Cd, Hg, Ni, Cu, Mo, Zn	P	-	-	NG	TDI, TWI, UL	Factor of RV	(4)
	iAs, Pb				G	BMDL	MOE	(9)
[246]	As	D	EDI	(1)	G	BMDL	MOE	(9)
[247]	As, Pb	P	EDI	(1)	G	BMDL	MOE and POE	(9) and 13
[248]	Pb	D	DDI	(1)	G	BMDL	MOE	(9)
[249]	Hg	P	I	(1) *	NG	RfD	THQ	(4) **

* When the exposure units are mg/day per person. ** When the exposure used is Equation (3).

Table 3 gives the results for mycotoxins, of which “nuts, nut products, and seeds” and “milk and dairy products” were the most studied foodstuffs, and are mainly from China and Iran (28%). The most studied age groups were adults (50.6%), followed by adolescents and children, and adults and children (around 12% in each age group). Almost all the authors opted for Equation (1) (91.5%) to calculate exposure. Equation (4) was used for non-genotoxic studies in half the cases, mainly using the TDI as the RV. For the genotoxic effect, 37% of the authors assessed the MOE (Equation (9)) and 19% the Hazard index (Equation (11)). In total, 7% assessed the cancer risk using Equation (14), and 35% assessed both the MOE and the cancer risk. 

**Table 3:** Exposure, damage, and risk characterisation methodology used in mycotoxin studies.

Reference	Mycotoxins	D/P	Exposure		Damage (Effect)		Risk Characterisation	
			Metric	Eq.	MOA	RV	Metric	Eq.
Beverage								
[250]	AFT	D	Exp ^a^	-	G	BMDL	MOE	(9)
[251]	AFT	D	EDI	(1)	G	TD50/Safety factor	HI	(11)
Cereals and bakery products								
[252]	FB1, FB2, FB3, DON	P	-	-	NG	PMTDI,	%PMTDI	(5)
	ZEA					TDI	%PMTDI	(5)
[253]	OTA, FB1, FB2, DON, NIV	P	-	-	NG	PMTDI, PTWI	HQ, HI	(4), (6)
	AFB1, AFB2, AFG1, AFM1	P	-	-	G	BMDL	MOE, MOET	(9), (10)
[254]	AFB1	P	CDI	(1)	G	BMDL	MOE	(9)
[255]	AF, OTA, DON	D	EDI	(1)	G	TD50/Safety factor	HI	(11)
[256]	AFB1	D	EDI	(1)	G	BMDL and PCP	MOE and CR	(9) and (14)
[257]	AFB1	D	Exp	(1)	G	BMDL and Pcancer	MOE and CR	(9) and (14)
[258]	DON, ZEN, OTA, TeA	D	EDI	(1)	NG	TDI, TTC	%HQ	(4)
	AME, AOH				G	TTC	HQ	(11)
[259]	AFB1, AFB2, AFG1, AFG2	D	EDI	(1)	G	TD50/Safety factor	HI	(11)
[260]	AFB1, AFB2, AFG1, AFG2	D	EDI	(1)	G	BMDL and Pcancer	MOE and Risk	(9) and (14)
[261]	ZEA, BEA, DAS, STER	D	EDI	(1)	NG	PMTDI	%HQ	(4)
	AFB1				G	AP	Risk	(14)
[262]	AFB1	D	APDI	(1)	G	BMDL and AP	MOE and cancer rate	(9) and (14)
[263]	FUM, OCHRA, DON	D	EDI	(1)	NG	TDI	HQ	(4)
	AFT				G	BMDL	MOE	(9)
[63]	AFB1, DON, OTA	P	y	(1)	NG	PTDI, PMTDI	MOS	(7)
Coffee, cocoa, and preparations								
[264]	OTA and FB2	D		(1)	NG	HBGV	%HBGV	(5)
	CIT, ENA, ENA1, ENB1, BEA	D	-	(1)	NG	HBGV, TTC	%HBGV and %TTC	(5) and (4)
	AME				G	TTC	%TTC	(11)
	AFB1, STC				G	BMDL	MOE	(9)
[265]	21 mycotoxins	D	EDI	(1)	NG	TDI, PTWI	%TDI	(5)
[266]	OTA, TENT, AME, AOH, ENB, ZEN	D	PDI	(1)	NG	TDI	% TDI	(5)
	OTA, AFs, STG				G	BMDL	MOE	(9)
[267]	OTA	P	CDI	(3)	NG	TDI	HQ	(4)
					G	BMDL	MOE	(9)
Eggs								
[268]	DON, ZEN	D	EDI	(1)	NG	TDI	HQ	(4)
	AFB1, OTA				G	BMDL	MOE	(9)
Fats and oils								
[142]	AFB1	P	Exp	-	G	BMDL and AP	MOE and Pr	(9) and (14)
Fruits, vegs, and legumes								
[269]	OTA	D	DDE	(1)	NG	PTWI	MOE	(7)
	AFB1				G	BMDL	MOE	(9)
[270]	Patulin	D	I	(2)	NG	PMTD	HQ	(4)
Tea, herbs and spices								
[226]	AFB1, TAF	D	EDI	(1)	G	BMDL	MOE	(9)
[271]	OTA and FB1	D	PDI	(1)	NG	TDI	%TDI	(5)
	AFB1				G	BMDL	MOE	(9)
[272]	HT2	D	PDI	(1)	NG	TDI	HQ	(4)
	AFB1, AFB2, TAF, OTA, STE				G	BMDL	MOE, MOET	(9), (10)
[273]	AFB1, AFB2, AFG1, AFG2	P	ADD	(1)	G	SF	R	(14)
[274]	AFB1, AFB2, AFG1, AFG2, ZEA	D	EDI	(1)	NG	TDI	%TDI	(5)
[275]	OTA, ZEN, DON, T-2, and FB	D	ADD	(1)	NG	RfD	HQ, HI	(4), (6)
	AFT	P	ADD	(1)	G	SF	R	(14)
Meat and meat products								
[276]	AFB1, AFB2, AFG1, AFG2	D	DE	(1)	G	BMDL	MOE	(9)
Milk and dairy products								
[277]	AFM1	D	EDI	(1)	NG	ISIRI	HI	(4)
					G	TD50/Safety factor	HI	(11)
[278]	AFM1	D	EDI	(1)	G	BMDL and Pcancer	MOE and Risk	(9) and (14)
[279]	AFM1	P	DE	(1)	G	BMDL and TD50/safety factor and Pcancer	MOE and HI and CR	(9) and (11) and (14)
[39]	AFM1	P	ADI	(1)	G	TD50/Safety factor and BMDL and CP	HI and MOE and LCR	(11) and (9) and (14)
[280]	AFM1	D	DE	(1)	G	BMDL and Pcancer and TD50/Safety factor	MOE and CR and HI	(9) and (14) and (11)
[281]	AFM1	P	EDI	(1)	NG	TDI	HI	(4)
	AFM1				G	TD50/Safety factor and Pcancer	HI and HCC	(11) and (14)
[282]	AFM1	D	EDI	(1)	G	BMDL and AP	MOE and risk	(9) and (14)
[283]	OTA	D	EDI	(1)	NG	PTWI	%PTWI	(5)
	AFM1				G	TD50/Safety factor	HI	(11)
	AFB1				G	BMDL	MOE	(9)
[284]	AFM1	D	EDI	(1)	G	TD50/Safety factor	HI	(11)
[285]	AFB1	D	EDI	(1) *	G	BMDL and Pcancer	MOE and CR	(9) and (14)
[40]	AFM1	P	EDI	(1)	G	BMDL and Pcancer and TD50/Safety factor	MOE and HCCrisk and HI	(9) and (14) and (11)
Miscellaneous								
[286]	OTA, OTB, FB1, FB2	D	EDI	(1)	NG	TDI, TWI	EDI/TDI	(4)
	AFB1				G	BMDL	MOE	(9)
[287]	DON, 3ADON, 15ADON, T-2, HT-2, NEO, NIV, ZEA, ENNB, ENNB1, ENNA, ENNA1, BEA, AFG2, OTA, DAS, βZAL.	D	PDI	(1)	NG	TDI	%TDI	(5)
[288]	OTA, FB1, ZEN	D	Exp	(1)	NG	PMTDI, PMTWI	%HBGV	(4)
	AFB1				G	BMDL and AP	MOE and PR	(9) and (14)
[289]	26 mycotoxins	D	APDI	(1)	G	BMDL and AP	MOE and LCR	(9) and (14)
[290]	AFB1, AFB2, AFG1, AFG2, AFT	D	PDI	(1)	G	BMDL	MOE	(9)
[291]	OTA	D	ADD	(3)	NG	RfD	HQ	(4)
[292]	AFM1, AFT	D	DE	(1)	G	BMDL and Pcancer	MOE and R	(9) and (14)
[293]	AFB1, AFB2, AFG1, AFG2	D	EDI	(1)	G	BMDL	MOE	(9)
[294]	AFB1, MCLR	P	DI	(1)	G	Toxicity factor	ORP	(9)
[295]	AFB1	D	EDI	(1)	G	BMDL and Pcancer	MOE and CR	(9) and (14)
[296]	FBs, OTA	D	PDI	(1)	NG	TDI, TWI	HBGV	(5)
	AFB1, AFT, BEA, CIT	D	PDI	(1)	G	BMDL	MOE	(9)
Nuts, nuts products and seeds								
[297]	AFB1	D	PDI	(1)	G	BMDL and AP	MOE and PR	(9) and (14)
[298]	OTA, FB1, FB2, ZEA, DON, 15AC-DON, 3AC-DON, T-2, HT-2	D	EDI	(1)	NG	TDI, PMTDI, PTWI	%TDI	(5)
[299]	AFB1, AFB2, AFG1, AFG2	D	ADD	(3)	G	BMDL	MOE	(9)
[300]	BEAU, CPA	D	PDI	(1)	NG	TDI	%TDI	(5)
	AFB1, AFT				G	BMDL	MOE	(9)
[301]	AFB1, AFB2, AFG1, AFG2, AFT, STC, BEA	D	EDI	(1)	NG	RfD	HQ	(4)
	AFB1, AFB2, AFG1, AFG2, AFT, STC				G	BMDL and AP	MOE and LCR	(9) and (14)
[302]	AFT	D	EDI	(1)	G	BMDL and AP	MOE and CR	(9) and (14)
[303]	AFT	P	DE	(1)	G	BMDL and Cancer potency	MOE and PR	(9) and (14)
Prepared dishes and snacks								
[304]	AFB1, AFT, OTA	D	DE	(1)	G	BMDL	MOE	(9)
[305]	AFB1, AFB2, AFG1, AFG2, OTA, ZEA, FB1, FB2, FUS, BEA, ENB, ENB1, ENA, ENA1	D	EDI	(1)	NG	TDI	%EDI-TDI	(5)
Total diet studies								
[306]	OTA	D	Exp	(1)	NG	PTWI	MOS	(7)
[307]	AFB1	P	EDI	(1)	G	BMDL, T25 and Pcancer	MOE and CR	(9) and (14)

* When the exposure units are mg/day per person. ^a^ Exp = Exposure.

Table 4 gives the results for acrylamide. The most often studied products were “cereals and bakery products” and “prepared dishes and snacks”. Most publications come from Iran (28.6%), followed by Lebanon and China (both with 9.52%). Adults were the most studied population group (38%), followed by adolescents and children (31%). Equation (1) was the most frequently used equation for exposure (71.4%). Equation (4) was used for 76.9% of the cases to characterise the non-genotoxic risk with RfD (69%) as the RV. The MOE was chosen for around 61.5% of the cases, and the cancer risk (Equation (14)) in 26.9% for the genotoxic effects. 

**Table 4:** Exposure, damage, and risk characterisation methodology used in acrylamide studies.

Reference	D/P	Exposure		Damage (Effect)		Risk Characterisation	
		Metric	Eq.	MOA	RV	Metric	Eq.
Cereals and bakery products							
[308]	D	CDI	(3)	NG	RfD	THQ	(4)
				G	OSF	CR	(14)
[309]	D	CDI	(3)	NG	RfD	THQ	(4)
				G	CSF	ILCR	(14)
[310]	P	CDI	(3)	NG	RfD	THQ	(4)
				G	BMDL and CSF	MOE and ILCR	(9) and (14)
Coffee, cocoa and preparations							
[311]	D	Y	(1)	NG	RfD	MOE	(7)
				G	BMDL	MOE	(9)
[312]	P	EDI	(2)	G	BMDL and PF	MOE and Risk	(9) and (14)
Fruits, vegs and legumes							
[313]	D	DE	(1)	G	BMDL	MOE	(9)
Tea, herbs and spices							
[314]	D	EDI	(1)	G	BMDL	MOE	(9)
Meat and meat products							
[315]	P	CDI	(3)	NG	RfD	THQ, HI	(4), (6)
				G	SF	ILCR	(14)
Miscellaneous							
[316]	P	E	(1)	NG	BMDL	MOE	(7)
				G	BMDL	MOE	(9)
[317]	D	ADD	(1)	NG	RfD	HI	(4)
				G	CPS	TR	(14)
[318]	D	E	(1)	G	BMDL	MOE	(9)
[319]	P	DE	(1)	G	BMDL	MOE	(9)
[320]	D	Y	(1)	G	SF	AC	(14)
[321]	P	DDE	(1)	G	BMDL	MOE	(9)
[322]	D	DE	(1)	G	BMDL	MOE	(9)
[323]	P	DE	(1)	NG	RfD	THQ	(4)
				G	BMDL and SF	MOE and ILCR	(9) and (14) **
[324]	P	CDI	(3)	NG	RfD	THQ	(4)
				G	CSF	ILCR	(14)
[325]	D	Exp	(1)	NG	RP	RPQ, RPI	(4), (6)
[326]	D	Exp	(1)	G	BMDL	MOE	(9)
Prepared dishes and snacks							
[327]	D	EDI	(1)	NG	TDI	HQ	(4)
				G	BMDL	MOE	(9)
[328]	D	DE	(1)	G	BMDL	MOE	(9)
[329]	D	DI	(1)	G	BMDL	MOE	(9)
[330]	D	EDI	-	NG	TDI	MOE	(7)
				G	BMDL, T25	MOE	(9)
[244]	P	DI	(1)	NG	NOAEL	MOE	(7)
				G	BMDL	MOE	(9)
[331]	P	EWI	(1)	NG	TWI	MOE	(7)
		Exp	(1)	G	BMDL	MOE	(9)
Total diet studies							
[332]	D	D	(1)	NG	NOAEL	MOE	(7)
				G	BMDL	MOE	(9)
[333]	D	EDI	(1)	G	CSF	ELCR	(14) **
[334]	D	E	(1)	NG	RfD		(5)
				G	BMDL	MOE	(9)

** When the exposure used is Equation (3).

Table 5 gives the PAH results. Most of the authors dealt with “fish and shellfish”. Iran (16%), Nigeria (14.3%), and China (12.5%) were the main countries of publication, while the adult population was the most studied group (65.5%). When calculating exposure, 68% of the studies opted for Equation (1). Equation (4) was applied to characterise the risk of non-genotoxic effects in all cases, using the RfD as RV in 93%. Equations (14) and (9), for genotoxic effects, were used in percentages of 43% and 29.4%, respectively.

**Table 5:** Exposure, damage, and risk characterisation methodology used in PAH studies.

Reference	PAH	D/P	Exposure		Damage (Effect)		Risk Characterisation	
			Metric	Eq.	MOA	RV	Metric	Eq.
Beekeeping products								
[126]	BaP, 4PAH, 8PAH, 16PAH and BaPeq	D	CDI	(3)	G	BMDL and CSF	MOE and ILCR	(9) and (14)
Cereals and bakery products								
[335]	BaP, 2PAH, 4PAH and 8PAH	D	EDI	(1)	G	BMDL	MOE	(9)
[136]	8PAH and BaPeq	D	E_D_	(1) *	G	SF	ILCR	(14) **
Fats and oils								
[336]	15PAH, 7PAH and BaPeq	D	-	-	G	SF	ILCR	(14) **
[337]	4PAH and BaPeq	D	DE	(1)	G	BMDL	MOE	(9)
[338]	16PAH and BaPeq	P	E_D_	(3)	G	SF	ILCR	(14)
[339]	15PAH and BaPeq	D	EDI	(1) *	G	SF	ILCR	(14) **
[340]	13PAH and BaPeq	P	CDI	(3)	G	BMDL and SF	MOE and ILCR	(9) and (14)
Fish and shellfish								
[341]	6PAH	D	EDDI	(1)	NG	RfD	THQ, HI	(4) **, (6)
	11PAH and BaPeq				G	SV and Q	HR and ILCR	(12) and (14) **
[342]	8PAH	D	-	-	NG	RfD	HI	(4) **
	7PAH and BaPeq	D	E_D_	(1) *	G	SF	ILCR	(14) **
[343]	8PAH	D	ADD	(2)	NG	RfD	HQ, HI	(4), (6)
	7PAH				G	SV and CSF	HR and Risk	(12) and (14)
[344]	4PAH	P	Exposure	(1)	G	BMDL	MOE	(9)
[151]	16PAH	D	-	-	G	LD50	HI	(11)
[345]	7PAH	D	EDI	(1)	NG	RfD	THQ	(4) **
	16PAH, BaPeq		EDI	(1)	G	CSF	CR	(14) **
[346]	BaP, 4PAH	D	DDE	(1)	G	BMDL	MOE	(9)
[347]	2PAH, 4PAH and 8PAH	D	EDI	(1)	G	BMDL	MOE	(9)
[348]	7 PAH	P	EDI	(1)	NG	RfD	HQ	(4)
	2PAH, 4PAH, 8PAH and BaPeq		-	-	G	BMDL and SF	MOE and ILCR	(9) and (14) **
[349]	8PAH and BaPeq	D	CDI	(3)	G	BMDL	MOE	(9)
[350]	16PAH and BaPeq	D	-	-	G	SV and CSF	HR and ILCR	(12) and (14) **
[351]	BaP and 4PAH	D	I	(1)	G	BMDL	MOE	(9)
[352]	7PAH	D	-	-	NG	RfD	THQ, HI	(4) **, (6)
[353]	BaPeq, 7PAH	D	EDI	(1)	NG	RfD	HQ	(4)
					G	OSF	CR	(14) **
[354]	4PAH, 8PAH, 16PAH and BaPeq	D	DDI	(1) *	G	BMDL and CSF	MOE and ILCR	(9) and (14) **
[355]	6PAH	D	I	(1)	NG	RfD	THQ	(4)
	6PAH				G	SF	TR	(14)
[356]	39PAH and BaPeq	P	CDI	(1)	NG	RfD	THQ, HI	(4) **, (6)
			EDI	(1)	G	SV and SF	HR and ILCR	(12) and (14) **
[357]	4PAH and BaPeq	D	DDI	(1) *	G	SV and SF	HR and ECR	(12) and (16)
[358]	6PAH	D	EDI	(1)	NG	RfD	THQ	(4) **
	7PAH and BaPeq				G	CSF	CR	(14) **
[359]	8PAH	D	-	-	NG	RfD	HQ	(4)
	16PAH andBaPeq				G	CSF	ILCR	(14) **
[360]	16PAH and BaPeq	D	-	-	G	SF	ILCR	(14) **
Fruits, vegs and legumes								
[96]	16PAH and BaPeq	P	CDI	(3)	G	BMDL and CSF	MOE and ILCR	(9) and (14)
[361]	15PAH and BaPeq	D	ADD	(3)	G	CSF	ILCR	(15)
Tea, herbs and spices								
[362]	16PAH	D	LADD	(3)	G	SF	RI, ƩRI	(14) ** and (16)
[363]	15PAH and BaPeq	P	-	-	G	CSF	R	(14) **
[364]	BaP, 2PAH and 4PAH	P	-	-	G	BMDL	MOE	(9)
Meat and meat products								
[365]	BaP, 4PAH and 8PAH	D	EDI	(1)	G	BMDL	MOE	(9)
[366]	8PAH, 14PAH and BaPeq	D	E_D_	(1) *	G	SF	ILCR	(14) **
[367]	BaPeq	D	LADD	(3)	G	CSF	CR	(14)
[368]	BaP and 4PAH	D	Exposure	(1)	G	BMDL	MOE	(9)
Milk and dairy products								
[369]	7PAH	P	ADD	(1)	NG	RfD	HQ, THQ	(4), (6)
	16PAH and BaPeq		CDI	(3)	G	CSF	ILCR	(14)
[370]	7PAH	P	ADD	(1)	NG	RfD	HQ, THQ	(4), (6)
	16PAH and BaPeq		CDI_BaP_	(3)	G	CSF	ILCR	(14)
[371]	16PAH and BaPeq	P	E_D_	(3)	G	BMDL and SF	MOE and ILCR	(9) and (14)
Miscellaneous								
[372]	16PAH and BaPeq	P	E_D_	(1) *	G	SF	ILCR	(15)
[373]	2PAH, 4PAH, BaP and BaPeq	P	EDI and E_D_	(1) and (1) *	G	BMDL and CSF	MOE and ILCR	(9) and (14) **
[374]	4PAH and BaPeq	D	EDI	(1)	G	BMDL	MOE	(9)
[375]	8PAH and BaPeq	D	DI	(1)	G	BMDL	MOE	(9)
[376]	8PAH and BaPeq	D	CDI	(3)	G	BMDL and BaP’s cancer risk	MOE and ILCR	(9) and (14)
[377]	16PAH and BaPeq	P	I	(1)	G	SF	CR	(14) **
[378]	4PAH, 16PAH and BaPeq	D	CDI	(3)	G	BMDL, BaP’s cancer risk	MOE and ILCR	(9) and (14)
[379]	7PAH	P	ADD	(1)	NG	RfD	HQ, THQ	(4), (6)
	16PAH, BaPeq	D	CDI	(3)	G	CSF_BaP_	ILCR, ILCR_act_	(14), (16)
[380]	BaP, 2PAH, 4PAH and 8PAH	D	DI	(1)	G	BMDL	MOE	(9)
[381]	16PAH and BaPeq	D	CDI	(3)	G	SF	CR	(14)
Total diet studies								
[334]	4PAH	D	E	(1)	G	BMDL	MOE	(9)

* When the exposure units are mg/day per person. ** When the exposure used is Equation (3).

#### 3.2.1. Exposure Assessment Equations and Nomenclature

Equation (1) was the formulation most used by the authors reviewed. Exposure was usually expressed as mg/kgBw/day, although the authors also used mg/day per person [382]. This second possibility is indicated in Table 1, Table 2, Table 3, Table 4 and Table 5 with an asterisk next to the equation number, i.e., (1*).

(1) $$\mathrm{Exposure}\;\left(\mathrm{mg}/\mathrm{kgBw}/\mathrm{day}\right)=\frac{\mathrm{Concentration}\;\mathrm{of}\;\mathrm{chemical}\;\mathrm{in}\;\mathrm{food}\;\left(\mathrm{mg}/\mathrm{kg}\right)\times \mathrm{Food}\;\mathrm{consumption}\left(\frac{\mathrm{kg}}{\mathrm{day}}\right)}{\mathrm{Body}\;\mathrm{weight}\;\left(\mathrm{kgBw}\right)}$$

The standard terminology (EHC 240) should be used for the consistent understanding and application of exposure. In this framework, Table 6 provides the different names used to designate exposure, while Table 7 gives the terminology used for the parameters in the equations, i.e., the concentration of the chemical hazard present in the food and food consumption, respectively. 

**Table 6:** Abbreviations and description of the terminology for exposure.

Parameter	Description	Parameter	Description
ADD	Average daily dose	EADI	Estimated average daily intake
ADI	Average daily intake	E_D_	Daily dietary exposure
AFE	Average food exposure	EDD	Estimated dietary doses
APDI	Average probable daily intake	EDDI	Estimated dietary daily intake
cEDI	Aggregated exposure	EDI	Estimated daily intake
CDI	Chronic daily intake	EDMI	Daily metal intakes
D	Total daily exposure	EWI	Estimated weekly intake
DC	Daily consumption	Exp	Exposure
DDE	Daily dietary exposure	Exposure	Dietary exposure
DDI	Dietary daily intake	I	Intake
DE	Dietary exposure	IEDI	International estimated daily intake
DED	Daily exposure dose	LADD	Lifetime average daily intake
DI	Dietary intake	NEDI	National estimated daily intake
DIM	Daily intake of metals	PDI	Probable daily intake
DIR	Daily intake rates	PEC	Potency equivalent concentration
E	Exposure/Total daily exposure	Y	Daily intake

**Table 7:** Abbreviations and description of concentrations and food consumption.

Parameter	Concentration Description	Parameter	Food Consumption Description
C	Concentration of chemical pollutants/residual concentration	AC	Average daily food consumption
CM	Average concentration	C	Estimated consumption of commodity
Cr	Concentration	CR	Consumption rate
R	Food pesticide residues	D	Daily intake
RC	Average residue concentration	F	Consumption of food/daily food consumption/food consumption rate
RL	Residue level	FER	Food eating rate
RLi	Occurrence of each residue	Fi	Average food consumption
STMR	Standard test of residual values	FIR	Daily intake
		I	Ingestion
		IR	Ingestion rate
		VIR	Daily ingested vegetable rate
		W	Average daily consumption

Some authors adapt the calculation of daily exposure by adding an adaptation factor to Equation (1) to convert it to Equation (2). This factor is intended to simulate, for example, the possible effect of process conditions on the variations in pesticide concentration present in food [59,72,75,90,383].

(2) $$\mathrm{Exposure}\;\left(\mathrm{mg}/\mathrm{kgBw}/\mathrm{day}\right)=\frac{\mathrm{Concentration}\;\mathrm{of}\;\mathrm{chemical}\;\mathrm{in}\;\mathrm{food}\;\left(\frac{\mathrm{mg}}{\mathrm{kg}}\right)\times \mathrm{Food}\;\mathrm{consumption}\;\left(\frac{\mathrm{kg}}{\mathrm{day}}\right)\times \mathrm{Adapting}\;\mathrm{factor}}{\mathrm{Body}\;\mathrm{weight}\;\left(\mathrm{kgBw}\right)}$$

The US Environmental Protection Agency recommends Equation (3) to estimate the average daily potential dose of an ingested contaminant through the consumption of food, water, soil, and dust [384]. In this case, the average daily exposure to a contaminant is the result of the total ingested concentration, measured in units of mass or volume per food, for example, in mg or L per kg food; the ingestion rate, e.g., the amount of contaminated food ingested by an individual during a given period expressed in units of mass or volume per unit time, such as kg/day or L/day; the duration of exposure or the amount of time an individual is exposed to the contaminant (e.g., years); and the frequency of exposure, e.g., in days per year, all divided by the average exposure time (e.g., days) and body weight (kgBw).

(3) $$\mathrm{Exposure}\;\left(\mathrm{mg}/\mathrm{kgBw}/\mathrm{day}\right)=\;\frac{Chemical\;concentration\;\left(\frac{\mathrm{mg}}{\mathrm{kg}}\right)\times Intake\;rate\;\left(\frac{\mathrm{kg}}{\mathrm{day}}\right)\times Exposure\;frequency\left(\frac{\mathrm{days}}{\mathrm{year}}\right)\times Exposure\;duration\left(\mathrm{years}\right)}{\mathrm{Body}\;\mathrm{weight}\;\left(\mathrm{kgBw}\right)\times \mathrm{Average}\;\mathrm{time}\;\left(\mathrm{days}\right)}$$

#### 3.2.2. Risk Characterisation Equations and Nomenclature

- Non-genotoxic chemical hazards

The risk ratio is the formula applied to characterise chemicals with a threshold level. The ratio is obtained by dividing the potential exposure to a non-genotoxic chemical hazard by the reference value at which no adverse effects are expected. The result is numerical but dimensionless and is considered a negligible risk when the value obtained is less than one. However, the ratio is also commonly expressed as a percentage obtained by multiplying the numerator of Equation (4) by 100. The risk ratio is given various names in the scientific literature (see Table 8).

(4) $$\mathrm{Risk}\;\mathrm{ratio}\;\left(\mathrm{non}-\mathrm{dimensional}\right)=\frac{\mathrm{Exposure}\;\left(\mathrm{mg}/\mathrm{kgBw}/\mathrm{day}\right)}{\mathrm{Reference}\;\mathrm{Value}\;\left(\mathrm{mg}/\mathrm{kgBw}/\mathrm{day}\right)}$$

**Table 8:** Abbreviations and description of risk ratio.

Parameter	Description	Parameter	Description
cHI	Consumer health risk	MOE	Margin of exposure
HHI	Health hazard index	MOS	Margin of safety
HI	Hazard index	R	Risk
HQ	Hazard quotation/hazard quotient	RQ	Risk quotient/Risk of ingestion
HRI	Hazard risk index/Health risk index	%RV *	Percentage of a reference value
IFS	Index of food safety	THQ	Target hazard quotient
		TS	Toxicity score

* E.g., % ADI, when RV is ADI.

Equation (5) is a particular case of Equation (4), where the RV is a HBGV (e.g., ADI, TDI, PTWI, etc.) and the chronic risk is expressed as a percentage (%HBGV).

(5) $$\%\mathrm{HBGV}\;\left(\mathrm{non}-\mathrm{dimensional}\right)=\frac{\mathrm{Exposure}\;\left(\mathrm{mg}/\mathrm{kgBw}/\mathrm{day}\right)}{\mathrm{HBGV}\;\left(\mathrm{mg}/\mathrm{kgBw}/\mathrm{day}\right)}\times 100$$

As the risk assessment of a single chemical is known to be insufficient, when the chemicals considered have the same MOA, the cumulative effect of multiple chemicals and multiple via routes should be considered, Equation (6) [121,385]. Table 9 gives the different terms used to define the cumulative risk.

(6) $$\mathrm{Cumulative}\;\mathrm{risk}\;\left(\mathrm{non}-\mathrm{dimensional}\right)=\sum_{i=1}^{N}Risk\;ratio\left(i\right)$$

**Table 9:** Abbreviations and descriptions used for cumulative risk.

Parameter	Description	Parameter	Description
cHI	Cumulative hazard index	THQ	Total hazard quotation
HI	Hazard index	TTHQ	Total target hazard quotient

The ratio for assessing the risk of non-genotoxic effects can also be obtained by calculating the margin of safety (MOS) (Equation (7)), where for similarity with the MOE equation, some authors use denomination MOE instead of MOS for non-genotoxic hazards [244,316].

(7) $$\mathrm{Margin}\;\mathrm{of}\;\mathrm{Safety}\;\left(\mathrm{non}-\mathrm{dimensional}\right)=\frac{\mathrm{Reference}\;\mathrm{value}\;\left(\mathrm{mg}/\mathrm{kgBw}/\mathrm{day}\right)}{\mathrm{Exposure}\;\left(\mathrm{mg}/\mathrm{kgBw}/\mathrm{day}\right)}$$

Doménech and Martorell [16] proposed the probabilistic safety margin (p_FSM) (Equation (8)), which represents the probability of exposure to a hazard *i* exceeding the safety limit (herein the ADIi), although this formulation can be extended to other RVs. The value obtained from this metric also lies between zero and one, so that a value close to one indicates a wide margin, i.e., exposure to this hazard is very unlikely to have consequences for health, while a margin close to zero implies a strong probability of a non-genotoxic adverse effect.

(8) $$p_{\mathrm{FSM}\left(H_{i}\right)}\;\left(\mathrm{non}-\mathrm{dimensional}\right)=\mathrm{Pr}\left\{\mathrm{HQ}\left(H_{i}\right)<1\right\}=\int_{0}^{{\mathrm{ADI}}_{i}}\mathrm{EDI}\left(H_{i}\right)\mathrm{dH}=1-\int_{{\mathrm{ADI}}_{i}}^{\infty }\mathrm{EDI}\left(H_{i}\right)\mathrm{dH}=1-\mathrm{EP}\left(H_{i}\right)$$

- Genotoxic chemical hazards

(a) Ratio metrics

To support risk management in hazards with genotoxic effects, the JECFA (Joint FAO/WHO Expert Committee on Food Additives) and the EFSA (European Food Safety Authority) proposed the margin of exposure (MOE) as the indicator of the level of deserved concern, Table 10. This approach makes no implicit assumptions of a “safe” intake and has been more widely used to assess substances that are both genotoxic and carcinogenic [14,31,386,387]. The MOE is quantified as the ratio between a defined RV for the adverse effect on the dose–response curve—generally, the BMDL% is related to a percentage increase in the response—and human exposure (Equation (9)) [388]. Equation (10) is used to assess the combined effect of substances with the same MOA.

(9) $$\mathrm{Margin}\;\mathrm{of}\;\mathrm{exposure}\;\left(\mathrm{non}-\mathrm{dimensional}\right)=\frac{\mathrm{Reference}\;\mathrm{value}\;\left(\mathrm{mg}/\mathrm{kgBw}/\mathrm{day}\right)}{\mathrm{Exposure}\;\left(\mathrm{mg}/\mathrm{kgBw}/\mathrm{day}\right)}$$

(10) $${\mathrm{MOE}}_{T}\left(\mathrm{non}-\mathrm{dimensional}\right)=\frac{1}{\frac{1}{\mathrm{MOE}1}+\frac{1}{\mathrm{MOE}2}+\frac{1}{\mathrm{MOE}3}+\ldots }$$

Alternatively, some authors use the hazard index metric or risk quotient for genotoxic effects (Equation (11)). This equation is very similar to the risk ratio (Equation (4)) proposed for non-genotoxic effects. In this case, the exposure is divided by a genotoxic reference value such as TD50/U.

(11) $$\mathrm{Hazard}\;\mathrm{index}\;\left(\mathrm{non}-\mathrm{dimensional}\right)=\frac{\mathrm{Exposure}\;\left(\mathrm{mg}/\mathrm{kgbw}/\mathrm{day}\right)}{\mathrm{Genotoxic}\;\mathrm{reference}\;\mathrm{value}\;\left(\mathrm{mg}/\mathrm{kgbw}/\mathrm{day}\right)}$$

The hazard ratio or the excess cancer risk can also be calculated to assess the margin for genotoxic hazards (Equation (12)). It can be estimated in terms of the incremental probability of developing cancer over a lifetime of total exposure to a potential carcinogen to humans. The cancer benchmark concentration is calculated by dividing the maximum acceptable risk level (1 × 10^−6^) by the slope factor, multiplying the value obtained by the body weight, and dividing this result by the consumption [57].

(12) $$\mathrm{Hazard}\;\mathrm{ratio}\;\left(\mathrm{non}-\mathrm{dimensional}\right)=\frac{\mathrm{Exposure}\;\left(\mathrm{mg}/\mathrm{kgbw}/\mathrm{day}\right)}{\mathrm{Cancer}\;\mathrm{benchmark}\;\mathrm{concentration}\;\left(\mathrm{mg}/\mathrm{kgbw}/\mathrm{day}\right)}$$

For the PAHs, the authors adapted Equation (12) through changing the exposure for the potency equivalent concentration values and dividing this value by the screening value (SV), calculated in the same way as the cancer benchmark concentration [343].

The POE is a complementary metric that represents the probability of the dose of exposure to a carcinogenic hazard exceeding the benchmark [247]. It is a measure of the probability—and, therefore, is dimensionless—of the change in the population’s response exceeding the predefined reference value. It could also be interpreted as the fraction of the total population exposed to an increased risk. One of the main advantages of the POE metric is that it considers the entire exposure distribution, represented with f(E) in Equation (13).

(13) $$\mathrm{POE}\;\left(\mathrm{non}-\mathrm{dimensional}\right)=\mathrm{Pr}\left(\mathrm{Exposure}>\mathrm{Reference}\;\mathrm{value}\right)=\int_{\mathrm{RV}}^{\infty }f\left(E\right)\;\mathrm{dE}$$

The POE metric is thus especially appropriate for characterising public health risks when the distribution of the exposure to a hazard with a genotoxic effect is positively skewed, and thus helps draw risk-informed conclusions, or for example if the MOE is below 10,000.

(b) Risk metrics

Different terms are used to estimate the cancer risks (see Table 10). This metric assesses the potential risk associated with exposure to carcinogens over a lifetime. It is obtained by multiplying the exposure by a slope factor (see Equation (14)). The slope factor is a toxicity value that quantifies a linear dose–response per mg/kgBw/day exposed, which is referenced to the abbreviations given in Table 11.

(14) $$\mathrm{Cancer}\;\mathrm{risk}\;\left(\mathrm{non}\_\mathrm{dimensional}\right)=\mathrm{Exposure}\;\left(\mathrm{mg}/\mathrm{kgBw}/\mathrm{day}\right)\;\times \;\mathrm{slope}\;\mathrm{factor}{\left(\mathrm{mg}/\mathrm{kgBw}/\mathrm{day}\right)}^{-1}$$

**Table 10:** Abbreviations and description for genotoxic effect.

Parameter	Description	Parameter	Description
AC	Annual number of cases	MOE	Margin of exposure
CR	Cancer risk	MOET	Total margin of exposure
CCR	Cumulative cancer risk	ORP	Overall risk probability
ECR	Excess cancer risk	POE	Probability of exceedance
ELCR	Excess lifetime cancer risk	PR	Population risk
HCC	Risk of hepatocellular carcinoma	R	Risk
HQ	Hazard quotation	Risk	Risk of cancer
HR	Hazard ratio	TCR	Total cancer risk/ Target cancer risk
ILCR	Incremental lifetime cancer risk	THQcarcinogenic	Target hazard quotient for carcinogenic risk
LCR	Lifetime cancer risk	TR	Total risk
LTCR	Lifetime cancer risk		

**Table 11:** Abbreviations and description of slope factor.

Parameter	Description	Parameter	Description
AP	Average potency	CSF	Cancer slope factor
BMC	Cancer benchmark concentration	OSF	Oral slope factor
CBC	Cancer benchmark concentration	Pcancer	Carcinogenic potency
CFS	Cancer slope factor	PCP	Population cancer potency
CPF	Cancer potency factor	Q	Oncogenic potency/BaP carcinogenic potency
CPSo	Oral cancer slope factor	SF	Slope Factor
CPS	Carcinogenic potency slope/carcinogens potency slope oral	PF	Potency factor

In the particular case of mycotoxins, the slope factor, also named carcinogenic potency (Pcancer), population cancer potency (PCP), or average potency (AP) were derived from a model with epidemiological data of individuals exposed to AFB1 testing positive for the hepatitis B surface antigen (HBsAg+) and testing negative for the hepatitis B surface antigen (HBsAg−) [389,390]. 

Some authors introduce an age-dependent adjustment factor to represent the increase in the probability of cancer from oral exposure, according to the population group, generally considered to be three for children and one for adults (see Equation (15)). Finally, the total cancer risk was assessed using Equation (16).

(15) $$\mathrm{Cancer}\;\mathrm{risk}\;\left(\mathrm{non}-\mathrm{dimensional}\right)=\;\mathrm{Exposure}\;\left(\mathrm{mg}/\mathrm{kgBw}/\mathrm{day}\right)\times \mathrm{Slope}\;\mathrm{factor}\;{\left(\mathrm{mg}/\mathrm{kgBw}/\mathrm{day}\right)}^{-1}\times \mathrm{Age}\_\mathrm{dependent}\;\mathrm{factor}$$

(16) $$\mathrm{Total}\;\mathrm{cancer}\;\mathrm{risk}\;\left(\mathrm{non}-\mathrm{dimensional}\right)=\sum_{i=1}^{N}\mathrm{Cancer}\;\mathrm{risk}\;\left(i\right)$$

#### 3.2.3. Harmonisation of the Terminology and Formulation

This work has shown how different authors use different terms and equations to define and formulate some of the parameters related to risk assessments. This section presents a proposal for terminology and formulation, considering the publications and guidelines recommended by some of the leading international organizations and their frequency of use in the literature review.

**Figure 3:**
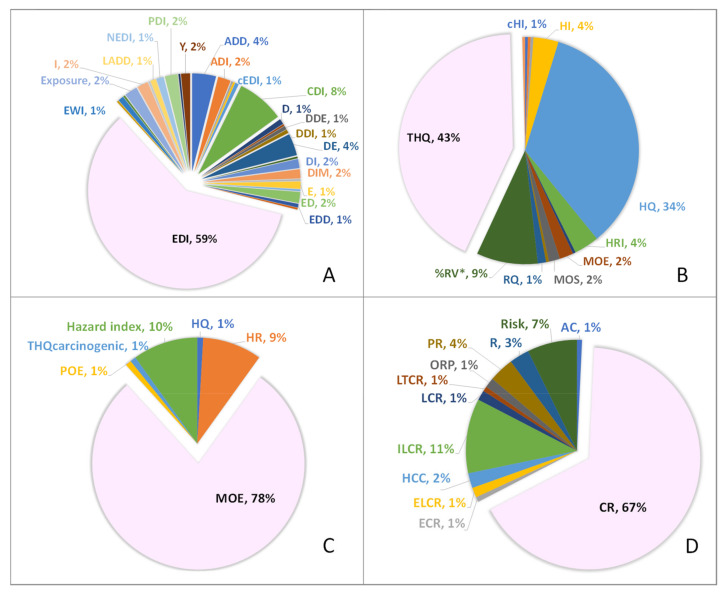
Metrics and terminology used by more than 1% of the authors reviewed: (**A**) exposure; (**B**) risk characterisation for non-genotoxic hazards; (**C**) risk characterisation for genotoxic hazards calculating a margin; and (**D**) calculating the risk.

The term most frequently used for exposure by the FDA [391], JECFA [392], EFSA [393] and CAC [394] is the EDI (estimated daily intake). As shown in Figure 3A, it is also the most reported term by the authors (59%), so it seems appropriate to recommend EDI for exposure. Concerning its formulation, Equation (3) is the most complete, and it is simplified to Equation (1) when the product of exposure frequency per the exposure duration is equal to the average time.

The metric to characterise the risk of non-genotoxic hazards suggested by EFSA [393], EPA [395] and ATSDR [396] is the ratio HQ (hazard quotient), which is obtained with Equation (4). However, Figure 3B shows that 43% of the reviewed articles used the target hazard quotient (THQ), compared to 34% that used the HQ. Nevertheless, it should be noted that most papers that used the THQ were related to the study of metals, whereas the HQ was used more on the other hazards studied. Therefore, HQ is the proposed terminology for general application to chemical hazards. On the other hand, since the international organisations mentioned and 71.8% of the papers have used the hazard index (HI) to estimate the cumulative effect of hazards, it seems that there is enough consensus on the use of this term and Equation (6) for its formulation. 

A margin and/or a risk can be calculated to characterise the risk of genotoxic hazards. In the first case, EFSA [386], EPA [395] and JECFA [397] among others, propose the margin of exposure (MOE), obtained with Equation (9). This formulation is also the ratio most used by the authors in the reviewed works (78%), Figure 3C. For this reason, the terms’ MOE for a single hazard and MOEt for the combined effect of several hazards with a genotoxic effect are proposed to assess the margin. This study also recommends combining the MOE with the POE, as the information they provide complements each other. On the other hand, international organisations such as ATSDR [27] use the term CR (cancer risk) to measure risk. This term is the one most frequently used by the authors (67%), Figure 3D, and, therefore, the one suggested to be found using Equation (14). In turn, in Equation (14), the slope factor is mostly referenced as CSF (cancer slope factor) in the studies and, therefore, the one proposed in this section. Finally, the term TCR (total cancer risk) and Equation (16) are recommended to calculate the cumulative risk of cancer.

## 4. Discussion

**Figure 4:**
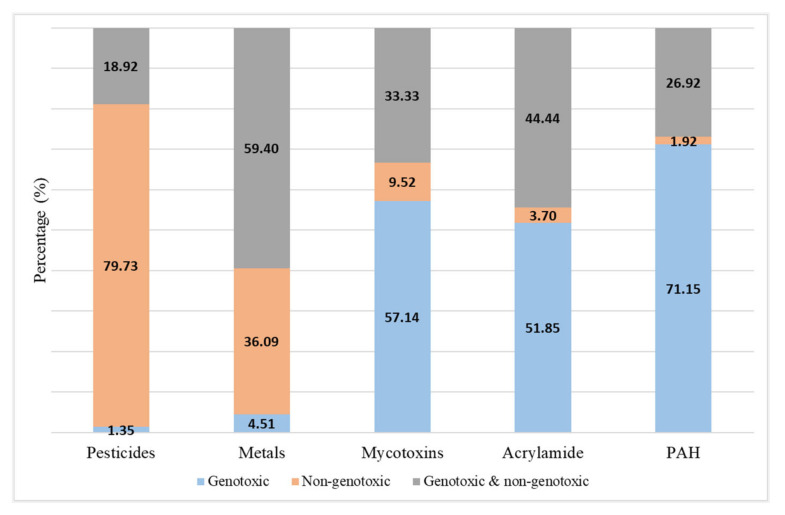
Percentage of studies considering the MOA per hazard, i.e., genotoxic, non-genotoxic, or the authors reviewed studied both effects.

Figure 4 shows the MOA considered to assess the risk characterisation for each studied hazard. In pesticides, most of the authors reviewed calculated the non-genotoxic effect (79.7%), while the study of the genotoxic and non-genotoxic effect (59.4%) was the preferred option for metals. However, only the genotoxic effect was studied for mycotoxins, acrylamide, and PAHs in more than half of the cases (57.14%, 51.8%, and 71.1%, respectively), Figure 4.

The findings show that the deterministic approach is the most frequently chosen option (see Table 6). This fact may be due to the advantages of this type of approach, such as its simple calculations and speed. Personnel do not need to be experts in risk analysis, and the results are usually sufficient for internal safety management [196]. However, for the genotoxic effects, the authors who assessed the risk characterisation of acrylamide, with the ratio and the risk, and the risk of mycotoxins, opted for a probabilistic approach with a higher percentage. An equal percentage was found in manuscripts that assessed the ratio and risk of metals. These findings may be related to the fact that more and more scientific papers need better realistic estimates that consider the entire distribution of model parameters. The main limitation here is that some input variables remain fixed in practice, so probabilistic and deterministic features appear in all models.

Focusing on hazards, 66.7% of the publications calculated the pesticide risk, and 33.3% assessed the ratio, Table 12. Risk was the most used option for metals (92.9%). The ratio was calculated in 58.9% of the publications on mycotoxins, while 37.5% calculated the ratio and the risk. In acrylamide, the ratio was calculated in 61.5% of the cases. The PAH risk was assessed using risk (43.1%), ratio (31.4%), and both metrics (25.5%).

**Table 12:** Percentage of publications considering the MOA, risk characterisation metric, hazard, and approach.

Hazard	Non-Genotoxic			Genotoxic								
	Ratio			Ratio and Risk			Ratio			Risk		
	%	%D	%P	%	%D	%P	%	%D	%P	%	%D	%P
Pesticides	100	83.6	16.4	-	-	-	33.3	100	-	66.7	60	40
Metals	100	82.7	17.3	2.4	50	50	4.7	75	25	92.9	83.5	16.5
Mycotoxins	100	81.5	18.5	37.5	71.4	28.6	58.9	83.9	16.1	7.1	25	75
Acrylamide	100	61.5	38.5	11.5	-	100	61.5	81.3	18.8	26.9	71.4	28.6
PAHs	100	80	13.3	25.5	61.5	38.5	31.4	87.5	12.5	43.1	72.7	27.3
Total	100	82	18	15.4	61.5	38.5	37.3	84.7	15.3	47.4	77	23

D = Deterministic approach and P = Probabilistic approach.

## 5. Conclusions

The development and application of risk assessment in different scientific fields worldwide has given rise to a wide variety of terms used for the same concepts. The present work analysed the terminology and formulations gathered from the field of risk characterisation of pesticides, metals, mycotoxins, acrylamide, and polycyclic aromatic hydrocarbons (PAHs), and reached the following conclusions.

The MOA of the chemical hazard determines the formulation used in risk characterisation. The ratio between the exposure and an RV is the only mathematical model used for non-genotoxic effects. This metric provides information on the level of concern. The most used ratios are the HQ for a single hazard and the HI for the cumulative effect of several hazards. For genotoxic effects, a margin and/or a risk can be calculated to characterise the risk. In the first case, the MOE is the author’s preferred metric in the literature review. Many studies highlight that using different RVs in the equations makes it difficult to compare the results. On the other hand, the metric adopted to characterise the risk of these genotoxic-chemical hazards is the cancer risk.

A deterministic approach is generally preferred to characterise risks, although differences can be found depending on hazards and metrics. Thus, a probabilistic approach is mainly used in the acrylamide articles when risk and ratio metrics are calculated. The same was true for mycotoxin studies when only a risk metric is calculated.

Based on the results found in the publications of international organisations and researchers, there appears to be a majority consensus on the parameters of risk characterisation and their formulation. This is why authors bring the following proposal for harmonisation: (1) exposure assessment is to be referred to as EDI (estimated daily intake); (2) the risk characterisation of a single non-genotoxic hazard uses the HQ (hazard quotient) metric and the HI (hazard index) for cumulative effect; (3) when a margin is used to characterise the risk of a single genotoxic hazard, the MOE (margin of exposure) metric combined with the POE (probability of exceedance) is to be selected, and when a risk metric is used in this context, the CR (cancer risk) measure is to be adopted, which, in turn, should be obtained using the CSF (cancer slope factor); (4) wherever possible, a probabilistic approach should be adopted for risk characterisation studies to take into account the effect of uncertainties in the quantification of parameters.

## Data Availability

No new data were created or analyzed in this study. Data sharing is not applicable to this article.
